# Aquifer Sustainability and the Use of Desalinated Seawater for Greenhouse Irrigation in the Campo de Níjar, Southeast Spain

**DOI:** 10.3390/ijerph16050898

**Published:** 2019-03-12

**Authors:** José A. Aznar-Sánchez, Luis J. Belmonte-Ureña, Juan F. Velasco-Muñoz, Diego L. Valera

**Affiliations:** 1 Department of Economics and Business, Research Centre CIAIMBITAL and CAESCG, University of Almería, 04120 Almería, Spain; lbelmont@ual.es (L.J.B.-U.); jfvelasco@ual.es (J.F.V.-M.); 2 Department of Engineering, Research Centre CIAMBITAL, University of Almería, Ctra. Sacramento s/n, 04120 Almería, Spain; dvalera@ual.es

**Keywords:** aquifers, sustainability, desalinated seawater, horticulture, cluster analysis, binary logistic regression

## Abstract

In the Campo de Níjar (southeast Spain), an intensive horticulture model under plastic has been developed based on the use of groundwater. For many years, aquifers have been overexploited, almost generating an environmental collapse. The construction of a desalination plant was planned to improve this situation and to achieve sustainable aquifer management. However, the aquifer is still being overexploited, since farmers scarcely use desalinated seawater for irrigation. In this paper, farmers irrigating with desalinated seawater are characterized, since they contribute to aquifer sustainability. The study aimed to identify the variables which condition the use of this water resource, as well as the kinds of incentives that encourage this option. For this purpose, a survey was undertaken within a sample of 110 farmers. A cluster analysis and a binary logistic regression were employed. The results from the cluster analysis allowed the characterization of farmers who use desalinated seawater for irrigation. Furthermore, the regression model showed the variables that determine a more intensive use of this irrigation source, such as crop diversification, availability of different water resources and the conductivity of aquifer water available for irrigation. The incentives promoting the use of desalinated seawater for irrigation that most encourage farmers are the implementation of tax relief, price reductions and the obligation to install rainwater collection systems.

## 1. Introduction

Aquifers are natural groundwater collectors of huge amounts of water [1]. Together with technological extraction improvements, the use of this water resource for human consumption and agriculture has notably increased in recent years all over the world [2,3]. Groundwater has especially been used in arid and semi-arid regions, where surface water is reduced, and its availability depends on the season [4,5]. Moreover, groundwater has some advantages for irrigation compared to surface water. The former provides continuous delivery, even in dry seasons, with no precipitation [6]. Irrigation based on groundwater provides reliable delivery and immediate access so that agriculture is not so vulnerable to droughts [2,5]. Apart from availability, extraction costs mean a reduced percentage out of the total production value. For this reason, a stable income flow is assured for the agricultural holding, as well as a higher stability of agriculture-related employment and a higher economic output when compared to agricultural holdings irrigated with surface water [2]. At the moment, it is estimated that agriculture accounts for 70% of total groundwater extraction all over the world [7].

Bad management and the intensive use of this resource, mainly for irrigation purposes, have brought about an overexploitation situation and the general depletion of aquifers [7,8,9]. The overexploitation of an aquifer is defined as when the total amount of water extracted is equal to or higher than the total amount of natural water recharge during a certain period of time [10,11]. In many arid and semi-arid regions, groundwater has been extracted at a rate far exceeding the aquifer recharge rate [12]. Aquifer overexploitation produces many negative environmental impacts, such as continuous lowering of the phreatic level, depletion of water resources, introduction of seawater into the aquifer, progressive worsening of water quality, an increase in extraction costs, reduction of agriculture production, land subsidence, desertification risk or impacts on surrounding ecosystems [5,9,13,14,15,16]. All of this jeopardizes not only the long-term sustainability of aquifers, but also the agricultural activity and the economic development of the region [6,10,17,18]. 

At the moment, efficient and sustainable use of aquifers has become an urgent priority, especially in those regions that are highly vulnerable to climate change, such as the Mediterranean basin [19]. As far as aquifers are concerned, Van Camp, Radfar and Walraevens [6] define sustainability as follows: “the level of development of groundwater that meets the needs of the present generation without compromising the ability of future generations to meet their needs”. According to Pongkijvorasin et al. [20], under an economic point of view, “water must be extracted such that its marginal benefit equals unit extraction cost plus the full marginal user cost, including the stock externality”. Many studies have analyzed aquifer sustainability and have proposed some management measures. Among them, we can find some management measures devoted to the water supply, such as the joint use of surface and groundwater [10,13,17,21], water transfers [22,23,24], the introduction of alternative water resources (desalinated or re-used water) [14,25,26,27,28], as well as the development of efficient rainwater collectors [29,30,31]. Regarding the demand side, the following proposals have been given: the limitation of groundwater extraction according to the natural water recharge level [15,32,33,34,35]; restrictions to irrigation and to new extractions [21,30,36]; rearrangements of water extraction points [18,37,38,39,40]; the establishment of charging systems in order to discourage well water consumption [19,41,42]; and efficiency improvements in water management [16,36,43,44].

This article analyses the use of desalinated seawater for greenhouse irrigation as an alternative option, in order to achieve aquifer sustainability. The study focuses on the Campo de Níjar region in Southeast Spain. In this area, intensive horticulture under plastic has been expanded based on groundwater irrigation [45,46]. The huge demand for groundwater for agriculture in this region has brought about negative impacts on aquifers [47]. On the one hand, continuous water extraction above the water recharge level has caused falls in piezometric levels. On the other hand, the quality of groundwater has worsened due to the introduction of seawater into the aquifer and the nitrate leaching coming from agricultural fertilizers [28,35]. The lack of alternative water resources and the expansion of cultivated surfaces under plastic almost made the aquifer level collapse. In this context, the Spanish government decided to build a desalination plant in 2004 aimed at providing an alternative water resource for irrigation in this region so that agricultural activity could continue and the aquifer sustainability could be assured. Some studies have concluded that if 50 percent of the irrigation needs are met with desalinated seawater, aquifer sustainability can be assured in this region [48,49]. It is therefore essential to get to know farmers who voluntarily decide to use desalinated seawater for irrigation and to determine which variables come into play for this decision. The objective of this paper is therefore two-fold: on the one hand, characterizing farmers and agricultural holdings with an intensive use of desalinated seawater for greenhouse irrigation; on the other hand, identifying variables which stimulate an intensive use of desalinated seawater for irrigation and analyzing the relationships among them. Based on this information, appropriate incentives can be identified in order to increase the use of desalinated seawater for irrigation and to contribute in this way to aquifer sustainability. For this purpose, a survey was conducted among a group of farmers who irrigate with desalinated seawater in the Campo de Níjar region. In order to analyze the primary data, a cluster analysis was undertaken, as well as a binary logistic model. The novelty of this research lies in applying this methodology to the analysis of non-conventional water resources for irrigation.

## 2. Materials and Methods

### 2.1. Study Region

**Figure 1:**
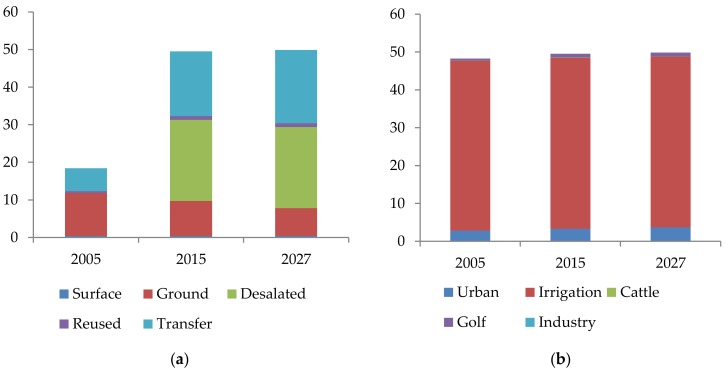
(**a**) Water sources in the basin of Campo de Níjar (hm^3^) *; (**b**) Demands of water from the basin of Campo de Níjar by sectors (hm^3^) *. * Adapted from the Hydrological Plan for the Andalusia Mediterranean Basin (Plan Hidrológico de la Demarcación Hidrográfica de las Cuencas Mediterráneas Andaluzas).

The Campo de Níjar region is located in the east of the Almería province in Andalusia, Spain. It is the second most important concentration of greenhouses in Almería, with a total of 5744 ha under plastic. This represents 18.6 percent of the total cultivated surface in greenhouses in the province [50]. This area suffers from the most acute water scarcity in the Mediterranean basin [51]. The Campo de Níjar region embraces a 780 km^2^ surface, where the agriculture production system of our study is located [52]. Figure 1 shows the hydric balance among available resources and demands in 2005, as well as the prospects of 2015 and 2027. In 2005, the main water supply was groundwater. Almost 93% of water demand was devoted to agriculture irrigation. There was, therefore, a relevant aquifer deficit of almost 30 hm^3^. In government planning, it was foreseen that this deficit was going to disappear through the use of 21.6 hm^3^ of desalinated seawater coming from the new desalination plant in Carboneras. However, the demand level of desalinated seawater for irrigation was much lower than foreseen, that is, under 10 hm^3^. This led to further overexploitation of the aquifer. 

**Figure 2:**
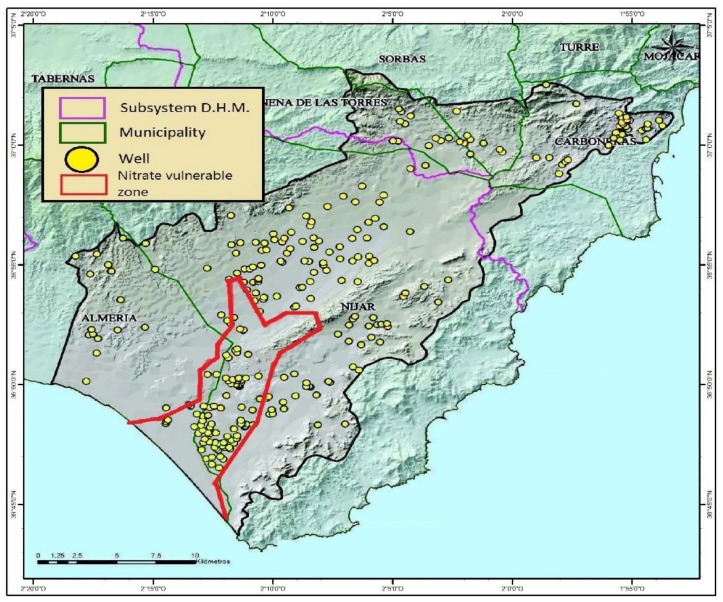
Extraction wells of the Campo de Níjar aquifer *. * Adapted from the Hydrological Plan for the Andalusia Mediterranean Basin (Plan Hidrológico de la Demarcación Hidrográfica de las Cuencas Mediterráneas Andaluzas).

The aquifer in the Campo de Níjar basin has a detritic nature with a surface of 582.02 km^2^ and an upwelling of 466.15 km^2^. Hereby, there is a 69.5 km^2^ surface that has been declared nitrate-vulnerable by the European Directive 91/676/CEE. The water extraction in this region is mainly done through wells, although some side exits to other water masses and to the sea can be observed (Figure 2). The aquifer recharge takes place through rainwater infiltration (56.5%), surface water (31.1%) and the feedback of agricultural irrigation (12.4%) [52]. 

In December 2004, the aquifer in the Campo de Níjar region was officially declared to have reached its overexploitation level. Apart from the poor quality level of this water mass, its bad chemical composition means a further problem with the use of this water. According to data from the Geological and Mining Institute of Spain [52], 47% of the aquifer control points registered an average content of chloride ion superior to 1000 mg/L. At some points, water measures have even reached 1770 mg/L and a conductivity of 7272 µS/cm. Measures undertaken by the Hydrographic District of the Andalusia Mediterranean Basin (Demarcación Hidrográfica de las Cuencas Mediterráneas Andaluzas) show concentration values of up to 2162 mg/L of chloride, up to 1175 mg/L of sodium and over 4 mg/L of boron at some points. These data clearly express the salinization process due to the intrusion of seawater as a consequence of its overexploitation level. As far as the nitrate concentration is concerned, the aquifer presents average values of almost 50 mg/L and some peaks superior to 300 mg/L. Other elements present with disturbing values are ammonium, nickel, fluoride, selenium, chromium, mercury, lead, arsenic, iron and manganese [52]. These data show that the water mass has been severely impacted by chemical alterations as a consequence of the strong agriculture pressure of the region. The following are reasons for the current poor aquifer quality: the inadequate use of fertilizers and agricultural phytosanitary products, deficiencies in the sanitation network, insufficient control of extractions, lack of future arrangement plans and lack of compliance with the existing ones, as well as a low level of irrigation with non-conventional sources of water. In this context of overexploitation and water pollution, it is estimated that the water resources of the aquifer will be exhausted in 10–15 years if the current trend is not reversed. Nevertheless, the reduction of the current extraction by up to 50% would guarantee natural recharge of the aquifer and thus the sustainability of the system [48].

### 2.2. Methodology

#### 2.2.1. Sample Selection

In order to characterize farmers who consume desalinated seawater in Campo de Níjar, a survey of the farmers associated with the Community of Water Users in Níjar (Comunidad de Usuarios de Aguas de la Comarca de Níjar—CUCN) was undertaken. The CUCN is a farmer association in charge of managing the water of the desalination plant in Carboneras in the Níjar District. Currently, it has a supply capacity of over 27 hm^3^ desalinated seawater per year. It provides irrigation water to 2200 farmers, who cultivate a land surface of 6800 ha in the districts of Almería, Sorbas and Níjar. The CUCN headquarters are located in the Níjar District where 1650 users are located with a cultivation surface of approximately 3318 ha. 

The sample was randomly selected among farmers associated with this irrigation community. Only minimal criteria were taken into account, such as crop type and usual cycle, and holding locations, so that the maximal heterogeneity regarding the socioeconomic and physical features of agricultural holdings were assured. The field research was carried out by the authors in September and October 2018. Each interview lasted around 25–35 min. The final number of valid interviews was 110. Due to the population size (3318 ha of cultivated land, irrigated with seawater) and the interviewed sample surface (363 ha), the error margin amounted to 4.85%.

#### 2.2.2. Data Analysis

The first objective of our data analysis was to determine whether there are relevant differences among the sampled farmers (110) who consume desalinated seawater for irrigation in Campo de Níjar. For this reason, the Density-Based Spatial Clustering of Applications with Noise (DBSCAN) algorithm was applied. Furthermore, this algorithm allowed us to find the optimal number of groups into which our sample can be divided. This methodology consists of a data mining technique that groups the subjects into homogeneous clusters regarding the criterion of observation density in a specific radius. This technique was proposed by Ester et al. [53] and has been recently applied to agriculture-related research [54,55,56,57].

Once relevant differences had been stated and the optimal number of groups was determined for the sample classification, the k-average classification algorithm was applied with the statistical software package SPSS (IBM SPSS Statistics version 23). For this purpose, the cluster number to be analyzed was introduced. The main advantage of the SPSS software for this analysis is that it allows identification of the agricultural holdings that have been grouped into a cluster. In this sense, it enables the cluster characterization regarding the classifying variables. Furthermore, the relevance level of each variable was observed; in other words, the variable relevance to build the groups of the sample observations. Among the variables, the most relevant ones for the study were selected: farmer’s academic level; main features and crop type of agricultural holdings; irrigation and fertilization techniques; farmers’ opinions regarding measures to improve the aquifer state and encouraging the use of desalinated seawater for irrigation. This kind of analysis is well known in environmental and agricultural studies [58,59,60]. 

Finally, once the sample of desalinated seawater users for irrigation in Campo de Níjar had been classified, the variables which have an impact on the consumption of desalinated seawater were analyzed. At this step, a binary logistic regression model was applied in order to analyze the results in terms of expectations. It also allowed identification of the variables that encourage higher consumption of desalinated seawater. This broadly applied technique requires the formulation of a dummy variable, which explains an event occurrence in terms of probability [61,62,63]. In this case, it was to reach a certain volume of consumed desalinated seawater. For our binary logistic regression model, a dependent dichotomous variable was supposed (V_0_) with two possible values (0 and 1), where V_1_, V_2_, … V_k_ was the set of independent variables observed in order to predict the V_0_ value. 

The probability relationship established by the binary logistic regression was the following:

$$Probability\;(V0=1|V1,\;V2,\;\ldots \mathrm{Vk})\;=\;\frac{1}{1+exp\left(-\beta 0-\beta 1v1-\beta 2v2-\ldots -\beta kvk\right)}.$$

The objective pursued was to calculate the parameter vector (β0, β1, β2…βk) that better suits the model. In this sense, in order to guarantee the goodness of fit, on a global scale, the Hosmer–Lemeshow test [64] was applied, which is provided by the statistical software SPSS.

## 3. Results and Discussion

### 3.1. Analysed Variables and Sample Characterisation

Table 1 gathers the descriptive statistical data of the analyzed variables within the research. Regarding the academic level of farmers, 8.2% of the interviewed farmers do not have a school degree, 62.6% have a compulsory school degree, 19.1% have an upper secondary school degree and only 10.1% possess a university degree. Regarding their insurance contracts, only 25.5% of the interviewed farmers contract insurance on the greenhouse structure, and less than 1% have fully comprehensive insurance over the agricultural holding. The average surface of a greenhouse is 1.3 ha, where the smallest one is 0.6 and the biggest one is 4.0 ha. The oldest greenhouse was built in 1986, and the newest one in 2014. The average greenhouse building year in our sample was 1999. 

The technological level of the irrigation system was characterized according to the existence of a dripping watering system, humidity control system and a watering programmer. A total of 14.6% of the agricultural holdings only use one of these three technologies, whereas 17.3% jointly use the three systems. The technological level of the greenhouse was estimated based on the structure type, the availability of activation systems for the side and roof windows and the availability of automatic ventilation and temperature control systems. A total of 73.6% of the greenhouses have a sloping roof construction, 25.5% are multi-tunnel constructions and under 1% are the flat-arch type. All greenhouses have windows, but only 15.5% have an automatic ventilation system. Only 1.8% of the agricultural holdings have heating systems, and 35.5% use a rainwater collection system. 

As far as the crop type is concerned, 42.7% of the cultivated land surface is devoted to a different crop than tomato, since this is the crop type that is most tolerant to high salinization levels of irrigation water. A total of 76.4% of the farmers interviewed repeat the crop type in the following seasons. The amount of available water resources for irrigation varies from one to three, apart from rainwater. These sources can be a community well with limited use, a private well with no limitations to water consumption and desalinated seawater. A total of 12.7% of farmers irrigate with only one source of water, 40.1% employ two water resources and 47.2% make use of the three different water resources to meet their irrigation needs. The quality of aquifer water, measured by its electrical conductivity level, ranges from 2.1 to 14 deciSiemens per meter (dS/m). The consumption of desalinated seawater covered, on average, 52.1% of the farmers’ irrigation needs, whereas this amounted to only 3% in the case of rainwater. A total of 1.8% of the interviewed farmers do not “setback” their land; this means that they do not create a fertilizing layer on the greenhouse soil and under the sanded surface layer. However, 17.3% of the farmers in the sample cultivate on such fertilized land, and 80.9% do it only partially.

During the survey, farmers were asked about their opinion on a set of measures that could be launched in order to improve the aquifer level and its conditions, as well as to foster the use of desalinated seawater for irrigation. A total of 52.7% agreed on the limitations set by the government to the well water extraction amount. A total of 50.9% of the farmers were positive regarding the price increase of well water. A total of 70.1% agreed on the closing up of illegal extraction wells. Only 3.7% were favorable to the prohibition of setting up new greenhouses. This measure was mostly refused. A total of 20.9% of the interviewed farmers would agree to the establishment of a minimum consumption of desalinated seawater for irrigation. A total of 99.1% would also agree to the compulsory establishment of rainwater collection systems, as well as to a price reduction of desalinated seawater. The best accepted measure is the establishment of tax relief for the consumption of desalinated seawater for irrigation. All farmers interviewed answered positively to this question. When asked about the continuity options for their agriculture exploitations, 40.1% of the farmers interviewed considered it possible that their descendants could still be undertaking agricultural activity in 20 years’ time.

### 3.2. Cluster Analysis

Table 2 shows the ANOVA cluster analysis undertaken in the survey sample according to the variables listed in Table 1. Fourteen out of the 23 variables were proven to be relevant in order to classify the sample. As a result, two homogeneous groups were built up with 49 and 61 agricultural holdings, respectively. The cluster 1 agglomerates consumers with low level use of desalinated seawater to irrigate their exploitations. Cluster 2 includes all farmers who consume at least 50% of desalinated seawater to satisfy their irrigation needs. It can be stated that these farmers contribute to aquifer sustainability, since their well water consumption allows the aquifer to naturally recharge. For this reason, the study of the significant classifying variables of the cluster allows characteristic exploitations that favor aquifer sustainability. These are shown in Table 3.

Farmers with a more intensive use of desalinated seawater for irrigation (cluster 2) have a higher level of education (upper secondary school) and a higher percentage of their agricultural holdings are insured. They also use modern irrigation systems and have a higher number of rainwater collectors. The intensive use of desalinated seawater allows them to diversify their crops, due to the lower water conductivity and salt concentrations in desalinated seawater. In cluster 1, the main crop type is tomato, since this crop type tolerates water salinity and conductivity the best, whereas the main crop type of cluster 2 is the pepper. 

The agricultural holdings in cluster 1 receive their irrigation water, on average, from two supplying water sources, but those in cluster 2 receive their irrigation water, on average, from three types of water supply. Regarding the use of non-conventional water sources, agricultural holdings in cluster 1 make use of 30.2% desalinated seawater and 0.2% rainwater to meet their irrigation needs. However, agricultural holdings in cluster 2 use 70.1% desalinated seawater and 4.3% rainwater. It is highly probable that the scarce irrigation with desalinated seawater in cluster 1 was due to the tomato monoculture and the relatively good quality of the available aquifer water. Its mean electrical conductivity is 2.8 dS/m, compared to 3.7 dS/m in cluster 2. As far as the fertilizing system is concerned, farmers in both clusters create a fertilizing layer on their cultivation soils (setting back technique) every 3 or 4 years. In cluster 1, this is done only partially, whereas in cluster 2, it is practiced all over the cultivated surface under plastic. 

Three of the proposed measures to make the aquifer exploitation more sustainable are relevant for the characterization of the clusters: extraction reduction, increasing the well water price and closing up illegal wells. However, some discrepancies can be found when comparing both clusters. Whereas 90% of the farmers in cluster 2 agree on it, farmers in cluster 1 showed a unanimous disagreement on these three measures. Regarding the compulsory establishment of a minimum consumption of desalinated seawater for irrigation, 4% of cluster 1 farmers agree on this measure compared to the 34% within cluster 2.

### 3.3. Logit Model

From a technical point of view, applying a binary logistic regression is proposed in order to identify independent variables that are useful to foresee the consumption trend of desalinated seawater for irrigation at a 50% rate, at least considering the total required water amount by each agricultural holding. Thus, as a dependent variable, a dichotomous variable (V_0_) was set where a value of 1 means at least 50% of seawater consumed for irrigation and 0 if the consumption level is inferior to this percentage. The goodness of the model is explained by Cox and Snell’s R^2^, as well as by Nagelkerke’s R^2^, which are normally used to justify the logistic regression goodness [65,66]. In this case, the independent variables included in the logistic regression would explain 65.1% to 93.6% of the dependent variable oscillation. In total, 96.4% of the cases are correctly classified. This model is therefore accepted to foresee the consumption of desalinated seawater of at least 50% to meet agricultural irrigation needs (V_0_). In this sense, the goodness-of-fit-analysis provided by the Hosmer–Lemeshow test is satisfactory, since the high p-value in this test is associated with the result of the dependent variable. A positive result of the test means the absence of significance (>0.05). In the study, the Hosmer–Lemeshow test gave a significance level of 0.814.

The result of the regression model shows that only three variables influence the consumption of desalinated seawater by at least 50%, which means they are associated with the sustainable use of water resources in the Campo de Níjar region according to the study hypothesis (Table 4). The relevant variables are the following: crop diversity (V_8_), number of available water resources (V_10_) and aquifer water conductivity (V_11_). 

In the Campo de Níjar, the main crop type cultivated is the tomato. This is due to the poor quality of water available for irrigation. The farmer is forced to cultivate a crop type that is tolerant to high water conductivity and salt concentration. In this sense, those farmers who wish to diversify production have to irrigate their crops with desalinated seawater, since the available well water does not reach a satisfactory level of quality. Variable V_8_ is directly related to the probability of consuming at least 50% desalinated seawater, since a value of 0 was given to the tomato crop and a value of 1 was given to any other crop type. The pepper crop type predominates in the cluster 2 greenhouses. This crop type is sensitive to water conductivity and salt. Desalinated seawater provides this crop type with the required quality of water for irrigation. 

Variables indicating the number of available water resources—at a consumption rate of at least 5% (V_10_)—have been proved to be significant and are directly related to higher consumption of desalinated seawater. The results from the cluster analysis showed that the agricultural holdings of cluster 2 have a higher number of available water resources for irrigation than the rest, apart from rainwater. This availability makes the water mixture possible, so that crops are provided with the required quality of irrigation water. This has been observed through the farmer’s logical evolution in the Campo de Níjar region, where desalinated seawater has been introduced to the irrigation mixture for agricultural holdings [35,51]. This option is opted for when desalinated seawater is available or when it is needed to reach an optimal water quality level. 

Aquifer water conductivity (V_11_) is relevant when explaining the priority of desalinated seawater consumption. This is due to the following reasons. Firstly, if the farmer wishes to diversify his/her crop production, a higher quality of irrigation water is required than that provided by well water. Secondly, it should be highlighted that water used by cluster 2 farmers presents a higher conductivity level (dS/m). As these farmers have access to aquifer water of poorer quality, they have no alternative to irrigating with desalinated seawater. Lastly, the cluster 2 farmers showed a higher level of knowledge and commitment regarding the poor aquifer conditions and the extraction impacts on the environment. For this reason, the construction of rainwater collectors is an additional way to reduce the overexploitation of the aquifer.

### 3.4. Measures to Assure Aquifer Sustainability

In the survey conducted, a set of measures to improve the aquifer conditions in the Campo de Níjar was researched. These measures included the following options: official public limitations to well water extraction, an increase in the well water price, closing up illegal wells, prohibition against building new greenhouses, obligation to consume a minimum amount of desalinated seawater for irrigation, obligation to install rainwater collection systems, reduction of the desalinated seawater price and the establishment of tax relief for the consumption of desalinated seawater for irrigation. 

The cluster analysis showed different answers from the two farmer groups regarding these options. Measures restricting the use of well water for irrigation were strongly refused by farmers with low consumption of desalinated seawater. This is obviously the group with higher potential to increase the use of alternative water resources for irrigation, since they mostly irrigate with well water. Measures achieving generalized acceptance were those which foster the higher use of alternative water resources. These are the establishment of tax relief for the consumption of desalinated seawater, the reduction of its price and the obligation to install rainwater collection systems. In the same way, the logit model results showed that these options are decisive for the consumption of desalinated seawater.

According to these results, a set of measures that improve the aquifer conditions can be proposed, and they would be, at the time, well accepted by farmers. First of all, the improvement of the water supply networks requires the springs available for farmers in the region to be increased. Apart from encouraging irrigation with desalinated seawater, this measure would provide better irrigation of water and higher diversification of crops. Secondly, current regulations should be modified so that the compulsory use of rainwater collectors in greenhouses is regulated. Thirdly, the establishment of tax relief for the consumption of desalinated seawater for irrigation is required. On the other hand, it can be inferred that the measure published by the public administration under the plan for the water districts in the Andalusia Mediterrenean Basin (Plan para la Demarcación Hidrográfica de las Cuencas Mediterráneas Andaluzas) which aims at the improvement and modernization of watering systems, is not useful in the study region, since the technological level of watering systems has been proved to be currently adequate and this variable does not have a relevant incidence on the consumption of desalinated seawater.

## 4. Conclusions

This article studied the use of desalinated seawater for greenhouse irrigation as an option to achieve aquifer sustainability in the Campo de Níjar region. The features of farmers irrigating with desalinated seawater were identified and analyzed; the variables with higher impact on the use of desalinated seawater were pinpointed and the most efficient incentives to increase the use of desalinated seawater for irrigation were discussed. In order to achieve these aims, comprehensive field research was conducted in a sample of 110 farmers made up of desalinated seawater users from the Campo de Níjar region. Data were analyzed through a cluster analysis and a binary logistic regression model.

The cluster analysis results allowed the characterization of farmers who irrigate more intensively with desalinated seawater, contributing, in this way, to aquifer sustainability. These farmers have a higher level of education than the rest of the farmers in the study region. Their agricultural holdings are equipped with modern watering systems and rainwater collection systems. They use different water resources for irrigation; on average, they use 70.1% desalinated seawater and 4.3% rainwater to meet their irrigation needs. The use of high-quality irrigation water allows them to diversify their crops.

The results from the binary logistic regression model have shed light on the decisive factors to allow more intensive irrigation with desalinated seawater; they are crop diversification, the availability of different irrigation water resources and the conductivity level of available aquifer water for irrigation. In light of these findings, the measures proposed to encourage irrigation with desalinated seawater and improve the aquifer state are as follows: improvement of the supply network with a higher number of available water springs, modification of current regulations regarding the compulsory installation of rainwater collectors in greenhouses and the establishment of tax relief for the consumption of desalinated seawater for irrigation. 

## Figures and Tables

**Table 1:** Descriptive statistics of the analyzed variables.

Variable	Description	Min	Max	Average	Standard Deviation	Variation Coefficient
V_1_	Farmer’s academic level	1	4	2.31	0.76	33.05%
V_2_	Contracted insurance	0	2	0.26	0.46	175.58%
V_3_	Greenhouse surface (ha)	0.60	4.00	1.29	0.43	33.53%
V_4_	Greenhouse building year	1986	2014	1999	4.50	0.23%
V_5_	Irrigation technological level	1	3	2.03	0.57	27.92%
V_6_	Greenhouse technological level	6	10	6.89	1.31	18.99%
V_7_	Rainwater collection system	0	1	-	-	-
V_8_	Crop diversity	0	1	-	-	-
V_9_	Monoculture level	1	4	2.65	0.98	36.95%
V_10_	Number of available water resources	1	3	2.35	0.70	29.69%
V_11_	Aquifer water conductivity level (dS/m)	2.1	14	3.33	2.24	67.32%
V_12_	Percentage of desalinated seawater use	0	1	0.52	0.25	48.22%
V_13_	Percentage of rainwater use	0	0.15	0.03	0.04	177.13%
V_14_	Setting back a fertilising layer on soil	1	5	3.72	0.93	25.01%
V_15_	Reduce water extraction	0	1	-	-	-
V_16_	Increase well water price	0	1	-	-	-
V_17_	Close illegal wells	0	1	-	-	-
V_18_	Prohibit construction of new greenhouses	0	1	-	-	-
V_19_	Establish the compulsory consumption of desalinated seawater	0	1	-	-	-
V_20_	Install a compulsory rainwater collection system	0	1	-	-	-
V_21_	Reduce desalinated seawater price	0	1	-	-	-
V_22_	Tax relief for the consumption of desalinated seawater	0	1	-	-	-
V_23_	Continuity of agricultural activity by descendants	0	1	-	-	-

**Table 2:** ANOVA analysis.

Variable	Description	Conglomerate Root Mean Square	df	Error Root Mean Square	df	F	*p*-Value
V_1_	Farmer’s academic level	7.364	1	0.520	108	14.169	0.000 *
V_2_	Contracted insurance	5.227	1	0.168	108	31.145	0.000 *
V_3_	Greenhouse surface (ha)	0.058	1	0.073	108	0.800	0.373
V_4_	Greenhouse building year	13.838	1	20.348	108	0.680	0.411
V_5_	Irrigation technological level	9.821	1	0.232	108	42.265	0.000 *
V_6_	Greenhouse technological level	0.259	1	1.726	108	0.150	0.699
V_7_	Rainwater collection system	9.865	1	0.142	108	69.603	0.000 *
V_8_	Crop diversity	9.346	1	0.163	108	57.445	0.000 *
V_9_	Monoculture level	0.158	1	0.970	108	0.163	0.687
V_10_	Number of available water resources	3.714	1	0.453	108	8.206	0.005 *
V_11_	Aquifer water conductivity level (dS/m)	23.451	1	4.853	108	4.833	0.030 *
V_12_	Percentage of desalinated seawater use	43,211.314	1	242.338	108	178.310	0.000 *
V_13_	Percentage of rainwater use	465.770	1	15.479	108	30.091	0.000 *
V_14_	Setting back a fertilizing layer on soil	22.651	1	0.663	108	34.160	0.000 *
V_15_	Reduce water extraction	24.566	1	0.026	108	930.109	0.000 *
V_16_	Increase well water price	22.901	1	0.043	108	538.822	0.000 *
V_17_	Close illegal wells	9.778	1	0.123	108	79.267	0.000 *
V_18_	Prohibit construction of new greenhouses	0.022	1	0.035	108	0.634	0.428
V_19_	Establish the compulsory consumption of desalinated seawater	2.502	1	0.145	108	17.224	0.000 *
V_20_	Install a compulsory rainwater collection system	0.007	1	0.009	108	0.802	0.373
V_21_	Reduce desalinated seawater price	0.007	1	0.009	108	0.802	0.373
V_22_	Tax relief for the consumption of desalinated seawater	0.000	1	0.000	108	-	-
V_23_	Continuity of agricultural activity by descendants	0.006	1	0.244	108	0.024	0.877

* 95% confidence significance.

**Table 3:** Cluster characterization of agriculture exploitations.

Variable	Description	Cluster 1 Lower Use of Desalinated Seawater	Cluster 2 Higher Use of Desalinated Seawater
V_1_	Farmer’s academic level	Upper secondary school	Upper secondary school/university
V_2_	Contracted insurance	2.0%	45.9%
V_5_	Irrigation technological level	1.7	2.3
V_7_	Rainwater collection system	2.0%	62.3%
V_8_	Crop diversity	Tomato or mixture	Pepper or mixture
V_10_	Number of available water resources	2	3
V_11_	Aquifer water conductivity level (dS/m)	2.8	3.7
V_12_	Percentage of desalinated seawater use	30.2%	70.1%
V_13_	Percentage of rainwater use	0.2%	4.3%
V_14_	Setting back a fertilizing layer on soil	Banding strips, 3–4 years	The whole surface, 3–4 years.
V_15_	Reduce water extractions	Completely disagree	95.1% stated “Generally agree or completely agree”
V_16_	Increase well water price	Completely disagree	91.8% stated “Generally agree or completely agree”
V_18_	Close illegal wells	Completely disagree	96.7% stated “Generally agree or completely agree”
V_19_	Establish the compulsory consumption of desalinated seawater	4.1% stated “Agree”	34.4% stated “Generally agree or completely agree”
	Total exploitations 110	49	61

**Table 4:** Variables of the logistic regression.

Variable	Description	β	Standard Error	Wald	fd	*p*-Value	Exp (β)
V_8_	Crop diversity	7.086	2.397	8.742	1	0.003 *	1195.422
V_10_	Number of available water resources	10.363	3.156	10.783	1	0.001 *	31661.706
V_11_	Aquifer water conductivity (dS/m)	2.125	0.865	6.036	1	0.014 *	8.370
Constant		‒31.308	10.363	9.127	1	0.003	0.000

* 95% confidence significance.
